# Pairing extinction training with vagus nerve stimulation reduces drug-seeking by altering activity in afferents to the medial prefrontal cortex

**DOI:** 10.3389/fnins.2026.1756644

**Published:** 2026-03-24

**Authors:** Christopher Driskill, Lily Vu, Sophia Jalilvand, Frank Salazar, Laney Waydick, Neha Suji, Sanjana Tata, Aamna Khan, Zarin Hasan, Ria Nuna, Zara Kanwal, Neissa Molin, Sven Kroener

**Affiliations:** Department of Neuroscience, University of Texas at Dallas, Richardson, TX, United States

**Keywords:** amygdala, cocaine, extinction, hippocampus, paraventricular nucleus of the thalamus, parvalbumin

## Abstract

**Introduction:**

Relapse triggered by drug-associated cues remains a major challenge in treating substance use disorders, as extinction learning is often weak and context dependent. Vagus nerve stimulation (VNS) enhances learning-related plasticity and, when paired with extinction training, reduces cue-induced reinstatement of cocaine seeking; however, the underlying circuit mechanisms remain unclear.

**Methods:**

We examined how VNS paired with extinction reshapes medial prefrontal cortex (mPFC) networks that regulate drug seeking, focusing on afferent inputs from the anterior (aPVT) and posterior (pPVT) paraventricular thalamus (PVT), posterior basolateral amygdala (BLA), and ventral hippocampus (vHPC). Using retrograde viral tracing combined with cFos immunolabeling, we identified pathway-specific effects of VNS on neurons projecting to the infralimbic (IL) and prelimbic (PL) cortex.

**Results:**

In the aPVT, VNS selectively increased activation of IL-projecting neurons, whereas in the pPVT it increased activity in PL-projecting neurons. VNS reduced overall BLA cFos expression, decreasing activation of BLA→IL projections while increasing BLA→PL activity. In the vHPC, VNS selectively reduced activation of IL-projecting neurons. Within the mPFC, VNS decreased overall neuronal activity but bidirectionally regulated parvalbumin interneurons, increasing their activity in PL and decreasing it in IL.

**Discussion:**

These findings show that pairing extinction with VNS remodels extinction circuits through projection- and cell-type–specific mechanisms, providing a framework for how VNS strengthens extinction learning and reduces relapse-like behavior.

## 1 Introduction

Re-exposure to drug-associated cues or stress reliably provokes craving and relapse in patients with substance use disorder (SUD) (O'Brien et al., 1998; Sinha et al., 2000). Extinction training reduces cue reactivity by removing the reinforcing consequences of drug use, but by itself it is often insufficient to affect permanent behavioral change, highlighting the need for strategies that enhance extinction memories to prevent relapse (Conklin and Tiffany, 2002; Taylor et al., 2009; Millan et al., 2011). Vagus nerve stimulation (VNS) is an FDA-approved treatment for epilepsy and depression (Rush et al., 2005; Nemeroff et al., 2006) that may be repurposed as an adjunct to exposure-based therapies for SUD. VNS induces the release of neuromodulators that promote cortical plasticity (Krahl and Clark, 2012; Cao et al., 2017), enhancing learning and memory in both rats (Clark et al., 1998; Cao et al., 2016; Driskill et al., 2022) and humans (Clark et al., 1999; Sun et al., 2017). In cocaine self-administering rats, pairing extinction with VNS facilitates extinction learning and reduces cue-induced reinstatement (Childs et al., 2017; Driskill et al., 2024), effects associated with altered activity in a circuit involving the medial prefrontal cortex, basolateral amygdala (BLA), and nucleus accumbens (NAc) (Childs et al., 2017; Arezoomandan et al., 2025).

In rodents the mPFC receives inputs from the paraventricular nucleus of the thalamus (PVT), the amygdala and the hippocampus, as well as other limbic structures, positioning it to integrate information regarding salience, value, and contextual cues associated with both appetitive and aversive outcomes (Peters et al., 2009; Gourley and Taylor, 2016). Within the mPFC, the prelimbic (PL) and infralimbic (IL) subregions are believed to serve largely opposing roles in regulating conditioned responses to both rewarding and aversive stimuli: The PL is implicated in the formation and expression of conditioned responses, and in the context of drug-seeking it drives reinstatement via its projection to the Nucleus accumbens core (NAc core). Conversely, the IL is important for the extinction of conditioned responses, inhibiting cocaine-seeking through its projections to the Nucleus accumbens shell (NAc shell) and/or to the PL (Peters et al., 2009; Peters et al., 2013; Muller Ewald et al., 2019). Here we further explored the network mechanisms through which VNS reduces drug-seeking behavior. We focused on VNS-induced changes in afferents to the mPFC from the PVT, BLA and the ventral hippocampus (vHPC), three areas that interact with the mPFC to regulate appetitive associative learning, memory retrieval, and extinction. Our goal was to determine whether pairing extinction training with VNS would differentially activate projections from these upstream regions to the PL and IL during reinstatement. To test this idea, we combined retrograde tracing from the IL and PL with immunohistochemical detection of cFos (Cruz et al., 2015) following cue-induced reinstatement in male rats that had received VNS or Sham-stimulation during extinction. In addition, in the mPFC we analyzed VNS-induced changes in cFos expression in parvalbumin-expressing interneurons (PVIs), which are targets of projections from the BLA and vHPC. Our results reveal a complex pattern of changes, with both global changes in cFos activity, as well as pathway-specific increases and decreases in projections to the IL and PL. In the mPFC, we found a decrease in cFos colabeling in PVI in the IL, as well as an opposite increase of cFos colabeling in PVI in the PL, indicating differential modulation of inhibition of mPFC networks by VNS. These results shed light on pathway-specific changes in the activation of areas that project to the mPFC that can be affected by pairing extinction with VNS.

## 2 Methods

### 2.1 Subjects

Male Sprague–Dawley rats (Taconic, Germantown, NY) were individually housed and kept on a 12-h. reverse light/dark cycle, with free access to food and water until surgery, when food was restricted to 25 g/day standard rat chow. At the time of surgery rats were at least 90 days old (250-300 g). All protocols were approved by the IACUC of The University of Texas at Dallas and were conducted in compliance with the NIH Guide for the Care and Use of Laboratory Animals.

### 2.2 Retrograde tracing

To visualize mPFC projections we infused a retrograde AAV expressing eGFP (*pENN.AAV.hSyn.HI.eGFP-Cre.WPRE.SV40*; 1 × 10^13^ vg/mL; Addgene viral prep #105540-AAVrg) bilaterally (0.4 μL each hemisphere, rate of 80 nL/min) into either the PL (+3.0 A/P, ±0.6 M/L, −3.9 D/V) or the IL (+3.0 A/P, ±0.6 M/L, −5.4 D/V) (Paxinos and Watson, 2007). Rats recovered for 7 days prior to VNS cuff and catheter implantation surgery.

### 2.3 Drug self-administration and extinction training

Drug self-administration and extinction training were performed as previously described (Childs et al., 2017; Driskill et al., 2024). Rats were anesthetized and implanted with a catheter in the right external jugular vein for drug administration. During the same surgery, a custom-made cuff electrode was placed around the left vagus nerve for the delivery of VNS (Childs et al., 2015). Seven days following surgery, rats were trained in a single overnight session to self-administer food pellets (45 mg, Bio Serv, Flemmington, NJ) in an operant conditioning chamber (Med Associates, Saint Albans, VT). Drug self-administration training took place in the same chamber, which was equipped with two levers, a house light, a cue light, and a tone. Each active lever press produced a 0.05 mL infusion of 2.0 mg/mL cocaine (NIDA Drug Program) in saline, and the presentation of drug-paired cues (illumination of the light over the active lever and the presentation of a 2,900 Hz tone), followed by a 20 s timeout. Self-administration sessions ended after 2 h. Both right and left levers were available for the duration of the session and drug-seeking behavior was quantified as active lever presses. Rats self-administered cocaine for 15–18 days, with a minimum criterion of at least 20 infusions per session. Subjects then underwent 10 days of extinction training in which lever presses on the previously active lever no longer produced cocaine or presentation of drug-paired cues. During extinction training rats received either sham-stimulation or non-contingent VNS (0.4 mA, 500 μs pulse width at 30 Hz, stimulation cycle of 30 s on, every 5 min) for the duration of the training session. After 10 days of extinction training, drug-seeking behavior was reinstated by presentation of the drug-associated cues in the operant conditioning chambers. During the reinstatement session presses on the previously active lever led to presentation of the drug-associated tone and light but did not result in drug delivery or VNS.

### 2.4 Immunohistochemistry

We used immunohistochemistry to colocalize AAVrg-eGFP labeled cells with the activity marker cFos in VNS- and Sham-stimulated rats following cue-induced reinstatement. Sixty minutes after the reinstatement session, rats were anesthetized with an overdose of urethane (3 g/kg i.p.) and transcardially perfused with room temp 1x PBS followed by 4% paraformaldehyde in 1x PBS (4 °C, pH 7.4). Brains were postfixed in PFA with 30% sucrose for 3 h. and were then transferred to 30% sucrose in PBS for approximately 18 h. at 4 °C. Coronal slices (40 μm) were cut on a freezing microtome and collected in PBS containing 0.01% NaN3 as a preservative. To determine VNS-induced changes in cFos expression and the colocalization of retrogradely labeled GFP + cells with cFos we analyzed the ventral subiculum and CA1 area of the ventral hippocampus in slices between −4.68 and −5.40 relative to bregma. Similarly, we analyzed cFos expression and GFP+/cFos colocalization in nuclei in the Basolateral Amygdala Complex, including the ventrolateral part of lateral nucleus (LaVL), the ventromedial part of lateral nucleus (LaVM), the anterior part of basolateral nucleus (BLa), and the posterior part of basolateral nucleus (BLp) in slices between bregma −2.52 and −3.24. These nuclei are referred here collectively as “BLA” and the results of their analyses were pooled together. We analyzed cFos expression and GFP+/cFos colocalization in the anterior paraventricular nucleus of the thalamus (aPVT) in slices between bregma −1.20 and −2.04. We analyzed cFos expression and GFP+/cFos colocalization in the posterior paraventricular nucleus (pPVT) in slices between bregma −3.00 and −4.08.

Free-floating sections containing BLA, pPVT, aPVT, and vHPC were incubated in guinea pig monoclonal recombinant anti-cFos (Synaptic Systems, Cat# 226308; RRID: AB_2905595; 1:10,000 working dilution) in PBS with 0.5% Triton and 2% normal goat serum (Thermo Fisher Scientific,) for 36 h. at 4 °C. Sections were washed at least 3 times for 10 min each in PBS before they were incubated in donkey monoclonal anti-Guinea pig Alexa 647 (Thermo Fisher Scientific, Cat# A-21450, RRID: AB_2535867, 1:5000 working dilution) for 2 h. at room temperature in 0.5% Triton-X and 2% normal goat serum in PBS. Sections were washed 3 times in PBS before they were mounted and cover-slipped using Prolong Gold Antifade with DAPI (Invitrogen, Grand Island, NY). For each animal a minimum of 4 sections of the mPFC, vHPC and BLA were imaged as z-stacks (3-micron step size) on a confocal microscope (FV3000, Olympus Corporation, Tokyo, Japan) with a 10x objective. Images were converted to Imaris file format and analyzed by an experimenter blind to the treatment conditions.

To assess changes in overall cFos expression and cFos expression within parvalbumin-expressing interneurons (PVIs), we analyzed coronal sections of the mPFC (IL and PL) spanning Bregma +3.72 to +2.52. cFos immunohistochemistry was performed as described above. For colabeling of cFos and parvalbumin, sections were incubated with a rabbit anti-PV primary antibody (Swant, Cat# PV27, RRID:AB_2631173; 1:2000 working dilution) together with the anti-cFos antibody during the primary incubation. Following washes, sections were incubated with a goat anti-rabbit Alexa Fluor 546 secondary antibody (Thermo Fisher Scientific, Cat# A-11010, RRID:AB_2534077; 1:1000 working dilution).

### 2.5 Data analysis

All statistical analyses were performed in GraphPad Prism 7.0.5 (GraphPad Software). We compared lever presses on the first day of extinction and during reinstatement with a one-way ANOVA, *Post hoc* analyses of main effects used Tukey’s multiple-comparison tests. Unpaired *t*-tests were used to compare differences in GFP + neurons, cFos+ neurons, and cFos expression in GFP + neurons. To compare changes in the mPFC in cFos+ neurons and cFos expression in PVIs we used separate two-way mixed effects ANOVAs with factors of treatment and region. *Post hoc* analysis was performed with a Holm–Šídák multiple comparisons test. Simple linear regression analysis with Holm–Šídák correction was conducted to assess correlations between PVI activity and active lever presses during reinstatement.

## 3 Results

### 3.1 Vagus nerve stimulation during extinction reduces cue-induced reinstatement

**Figure 1:**
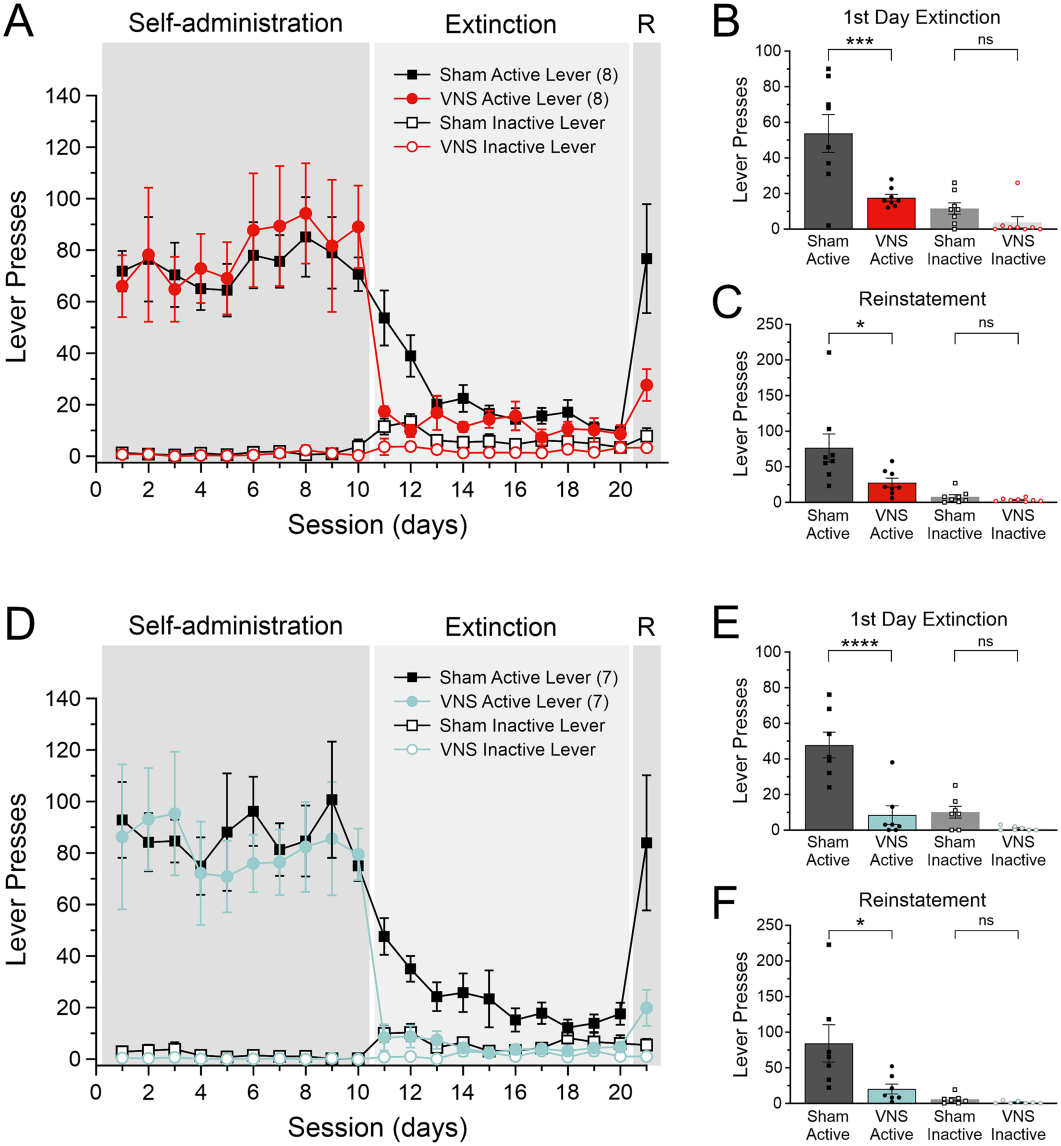
Vagus nerve stimulation (VNS) facilitates extinction from cocaine seeking and reduces cue-induced reinstatement. **(A)** Active (solid symbols) and inactive lever presses (open symbols) in rats that received infusions of a retrograde AAV into the infralimbic cortex (IL) during cocaine self-administration, extinction, and cue-induced reinstatement (R). Rats received either VNS (red symbols, *n* = 8) or sham stimulation (black symbols, *n* = 8) during extinction on days 11–20. **(B)** VNS-treated rats displayed reduced active lever presses during the first day of extinction. **(C)** Responses on the previously active lever during cue-induced reinstatement are significantly reduced in VNS-treated rats. **(D)** Active (solid symbols) and inactive lever presses (open symbols) in rats that received infusions of the retrograde AAV into the prelimbic cortex (PL) during cocaine self-administration, extinction, and cue-induced reinstatement (R). Rats in this cohort also received either VNS (Teal symbols, *n* = 7) or sham-stimulation (Black symbols, *n* = 7) during extinction training. **(E)** VNS-treated rats with PL infusions also showed accelerated extinction on the first day of extinction, **(F)** and reduced responding at the active lever during cue-induced reinstatement. *p* values are (*) < 0.05, (***) < 0.001, and (****) < 0.0001.

Separate groups of rats received infusions of retrograde AAV expressing eGFP into either the infralimbic cortex (IL) or prelimbic cortex (PL). These rats were then trained to self-administer cocaine for 15–18 days, followed by 10 days of extinction training paired with either vagus nerve stimulation (VNS) or Sham stimulation. Twenty-four hours after the last day of extinction training, drug-seeking behavior was reinstated in a cued reinstatement session by presenting the conditioned drug cues (Figure 1). Figure 1A shows lever presses during the last 10 days of drug-self administration, during the 10 days of extinction, and the reinstatement session in rats that received retro-eGFP infusions into the IL. We compared behavior between rats that received Sham stimulation (*n* = 8) or VNS (*n* = 8) during the extinction period. A one-way ANOVA found a significant effect of treatment on lever presses during the first day of extinction [*F*_(3, 28)_ = 14.24, *p* < 0.0001, Figure 1B]. *Post hoc* analysis with Tukey’s multiple comparisons test showed a significant decrease in active lever presses (*p* = 0.0009), but no difference in the number of inactive lever presses (*p* = 0.7856). Similarly, a one-way ANOVA for responses during the cue-induced reinstatement session found a significant effect of treatment on lever presses [*F*_(3,28)_ = 9.087, *p* = 0.0002, Figure 1C]. Post hoc analysis with Tukey’s multiple comparisons test showed a significant decrease in active lever presses in animals that received VNS (*p* = 0.0212), but no difference in the number of inactive lever presses (*p* = 0.9923). We performed the same analyses for drug-seeking behavior during the extinction period and the reinstatement session, respectively, in rats that received an AAV infusion of retro-eGFP into the PL (Figure 1D) comparing lever presses between rats that received VNS (*n* = 7) or Sham-stimulation (*n* = 7). A one-way ANOVA found a significant effect of treatment on lever presses during the first day of extinction [*F*_(3, 24)_ = 19.58, *p* < 0.0001, Figure 1E). *Post hoc* analysis with Tukey’s multiple comparisons test showed a significant decrease in active lever presses in animals that received VNS (*p* < 0.0001), but no difference in the number of inactive lever presses (*p* = 0.5350). A one-way ANOVA for lever presses during the cue-induced reinstatement session found a significant effect of treatment [*F*_(3,24)_ = 7.925, *p* = 0.0008, Figure 1F]. Post hoc analysis with Tukey’s multiple comparisons test showed a significant decrease in active lever presses in animals that received VNS (*p* = 0.0143), but no difference in the number of inactive lever presses (*p* = 0.9956).

### 3.2 Distribution of neurons projecting to the mPFC

**Figure 2:**
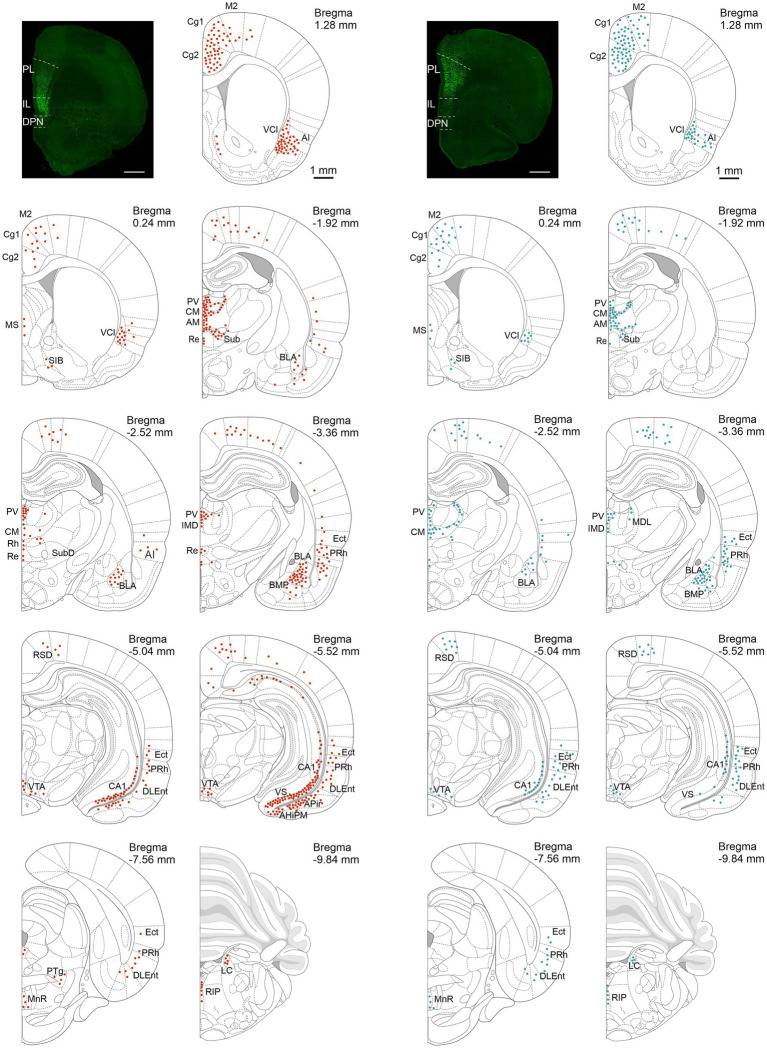
Photomicrographs of infusions sites of a retrograde AAV expressing eGFP in the infralimbic cortex (IL, left) and the prelimbic cortex (PL, right) and corresponding distribution of retrogradely labeled cells in major brain nuclei (IL, red symbols; PL teal symbols). Each dot represents about 5–10 cells; averages for 4 rats (2 Sham and 2 VNS) for each infusion site. Abbreviations: AHiPM, Amygdalohippocampal area, posteromedial part; Ai, Agranular insular cortex; AM, Anteromedial thalamic nucleus; APir, amygdalopiriform transition area; BLA, basolateral amygdala complex; BMP, basomedial amygdaloid nucleus, posterior part; CA1, cornu ammonis area 1 of the hippocampus; Cg1, cingulate cortex, area 1; Cg2, cingulate cortex, area 2; CM, Central medial thalamic nucleus; DLEnt, dorsolateral entorhinal cortex; Ect, ectorhinal cortex; IMD, intermediodorsal thalamic nucleus; LC, locus coeruleus; M2, secondary motor cortex; MDL, mediodorsal thalamic nucleus, lateral part; MnR, median raphe nucleus; MS, medial septum; PRh, perirhinal cortex; PTg, pedunculotegmental nucleus; PV, paraventricular thalamic nucleus; Re, reuniens thalamic nucleus; RIP, raphe interpositus nucleus; RSD, retrosplenial dysgranular cortex; SiB, substantia innominata, basal part; Sub, submedius thalamic nucleus; VCl, ventral part of claustrum; VS, ventral subiculum; VTA, ventral tegmental area.

Figure 2 shows the brain-wide distribution of retrogradely eGFP-labeled cells in major nuclei of the rat brain that project to either the IL (red symbols) or the PL (teal symbols). For each infusion group (PL or IL) we took sections from four rats (2 VNS- and 2 Sham-treated rats each) to map out the distribution of eGFP-expressing neurons. For each brain region we took the average number of eGFP-expressing cells across the four brains and placed one dot for approximately every 5–10 cells. Significant numbers of cells were found in midline thalamic nuclei, the BLA, the vHPC, and the major sources of neuromodulatory inputs to the mPFC, including the ventral tegmental area (VTA), midline raphe nuclei, and the locus coeruleus (LC). In this report we focus on VNS modulation of the projections from the PVT, the BLA, and the CA1 region of the vHPC.

### 3.3 Effect of VNS on paraventricular nucleus of the thalamus to the mPFC

**Figure 3:**
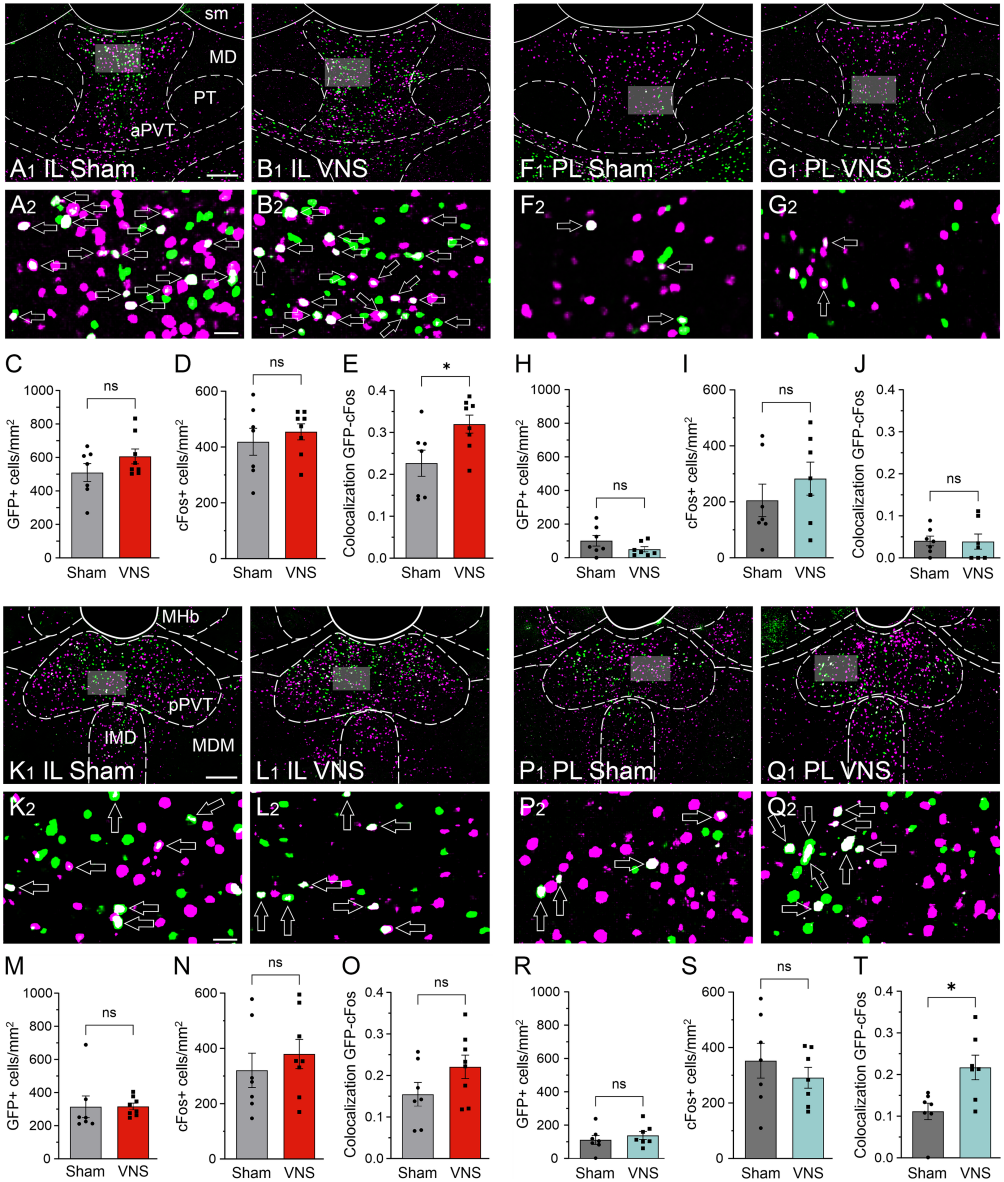
VNS differentially modulates cFos expression following reinstatement in IL- and PL-projecting neurons of the paraventricular nucleus of the thalamus (PVT). Panels A-J show representative images and analysis of GFP + and cFos+ cells in the anterior PVT (aPVT) of Sham- and VNS-treated rats, while panels K-T show examples and analyses for the posterior PVT (pPVT). **(A,B)** GFP-positive cells (green) following infusion of a retro-AAV into the IL and cFos (pink) in the aPVT of rats that received either Sham-stimulation (*n* = 8), **(A)** or VNS (*n* = 8) during extinction **(B)**. **(A_**2,**_,B_**2,**_)** Arrows indicate GFP + cells colocalized with cFos (white) in a magnified view of the area indicated by shading in (**A_**1,**_,B_**1,**_)**. (**C,D)** VNS and Sham-stimulated rats did not differ in the number of GFP + cells **(C)** or cFos+ **(D)** in the aPVT. **(E)** VNS increased reduced cFos expression in IL-projecting (GFP+) neurons. **(F,G)** GFP-positive cells following infusion of the retro-AAV into the PL and cFos in the aPVT of rats receiving either Sham-stimulation (*n* = 7) **(F)**, or VNS (*n* = 7) during extinction **(G)**. **(H–J)** VNS and Sham-stimulated rats did not differ in the number of GFP + or cFos+, as well as GFP+/cFos+ colocalized cells in the aPVT. **(K,L)** GFP-positive cells following infusion of a retro-AAV into the IL and cFos in the pPVT of rats that received either Sham-stimulation **(K)** or VNS **(L)** during extinction. **(K**_**2**_**,L**_**2**_**)** Arrows indicate GFP + cells colocalized with cFos (white). **(M–O)** VNS and Sham-stimulated rats did not differ in the number of GFP + or cFos+, as well as GFP+/cFos+ colocalized cells in the pPVT. **(P,Q)** GFP-positive cells following infusion of the retro-AAV into the PL and cFos in the pPVT of rats receiving either Sham-stimulation or VNS during extinction. **(R,S)** VNS and Sham-stimulated rats did not differ in the number of GFP + or cFos+ cells; however, the number of cFos+ cells projecting to the PL was significantly increased by VNS **(T)**. Scale bars represent 200 μm in **(A**_**1**_**,B**_**1**_**,F**_**1,**_**G**_**1**_**,K**_**1**_**,L**_**1**_**,P**_**1,**_**Q**_**1**_**)**, 25 μm in **(A**_**2**_**,B**_**2**_**,F**_**2,**_**G**_**2**_**,K**_**2**_**,L**_**2**_**,P**_**2,**_**Q**_**2**_**)**. *p* values are (*) < 0.05.

In rats that received retro-AAV-eGFP infusions into the IL, we quantified cFos expression and GFP+/cFos colocalization in the aPVT and pPVT to assess VNS-induced changes in the activation of IL-projecting neurons (Figure 3). In the aPVT, separate unpaired t-tests revealed no difference in the number of GFP + cells [t_(13)_ = 1.396, *p* = 0.1862, Figure 3C] and no difference in the total number of cFos+ cells [t_(13)_ = 0.6536, *p* = 0.5248, Figure 3D], but a significant increase in the percentage of GFP + cells expressing cFos [t_(13)_ = 2.515, *p* = 0.0259, Figure 3E] in VNS-treated (*n* = 8) compared to Sham-treated rats (*n* = 7). In the pPVT, separate unpaired t-tests showed no difference in the number of GFP + cells [t_(13)_ = 0.03269, *p* = 0.9744, Figure 3M], no difference in total cFos+ cells [t_(13)_ = 0.7267, *p* = 0.4803, Figure 3N], and no difference in the percentage of GFP + cells expressing cFos [t_(13)_ = 1.641, *p* = 0.1248, Figure 3O] between groups. We performed the same analyses in rats that received retro-AAV-eGFP infusions into the PL (Figure 3). In the aPVT, separate unpaired t-tests revealed no difference in the number of GFP + cells [t_(12)_ = 1.431, *p* = 0.1780, Figure 3H], no difference in total cFos+ cells [t_(12)_ = 0.9371, *p* = 0.3672, Figure 3I], and no difference in the percentage of GFP + cells expressing cFos [t(12) = 0.07161, *p* = 0.9441, Figure 3J] between Sham-treated (*n* = 7) and VNS-treated rats (*n* = 7). In contrast, in the pPVT, there was no difference in the number of GFP + cells [t_(12)_ = 0.7400, *p* = 0.4736, Figure 3R] or total cFos+ cells [t_(12)_ = 0.8373, *p* = 0.4188, Figure 3S], but a significant increase in the percentage of GFP + cells expressing cFos in VNS-treated rats [t_(12)_ = 2.973, *p* = 0.0116, Figure 3T]. Taken together, these results indicate that VNS selectively enhances recruitment of the aPVT➔IL and pPVT➔PL circuits without altering overall PVT activation.

### 3.4 Effect of VNS on projections from the BLA to the mPFC

**Figure 4:**
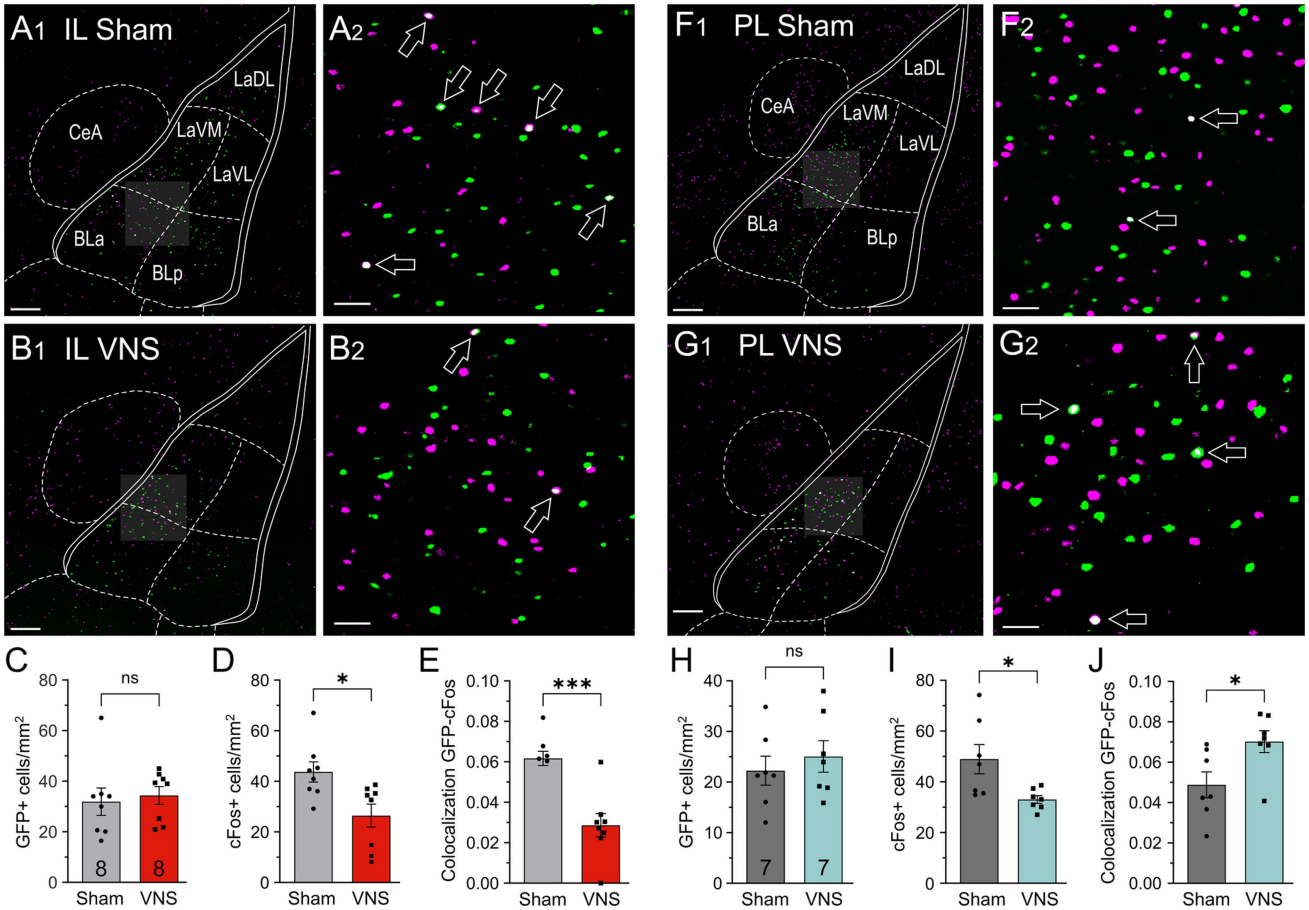
VNS differentially modulates cFos expression following reinstatement in IL- and PL-projecting neurons of the basolateral amygdala (BLA). **(A,B)** GFP-positive cells (green) following infusion of a retro-AAV into the IL and cFos (pink) in the BLA of rats that received either Sham-stimulation (*n* = 8), **(A)** or VNS (*n* = 8) during extinction **(B)**. **(A**_**2**_**,B**_**2**_**)**. Arrows indicate GFP + cells colocalized with cFos (white). **(C)** VNS and Sham-stimulated rats did not differ in the number of GFP + cells in the BLA. **(D)** VNS reduced overall cFos expression in the BLA. **(E)** VNS also reduced cFos expression in IL-projecting (GFP+) neurons. **(F,G)** GFP-positive cells following infusion of the retro-AAV into the PL and cFos in the BLA of rats receiving either Sham-stimulation (*n* = 7) **(F)**, or VNS (*n* = 7) during extinction **(G)**. **(H)** VNS and Sham-stimulated rats did not differ in the number of GFP + cells in the BLA. **(I)** VNS also reduced overall cFos expression in the BLA in this cohort. **(J)** In contrast to its effect on IL-projecting neurons, VNS selectively increased cFos labeling in PL-projecting neurons. Scale bars represent 200 μm in **(A_**1,**_,B_**1,**_,F_**1,**_,G_**1,**_)**, and 100 μm in **(A_**2,**_,B_**2,**_,F_**2,**_,G_**2,**_)**. *p* values are (*) < 0.05, and (***) < 0.001.

We analyzed cFos expression during drug-seeking and colocalization of cFos in IL-projecting neurons in the basolateral complex in brains from the same rats receiving VNS (*n* = 8) or Sham-stimulation (*n* = 8) (Figures 4A–E). Separate unpaired t-tests showed no difference in the number of GFP + cells [t_(14)_ = 0.3803, *p* = 0.7094, Figure 4C]; however, there was a significant overall decrease in cFos+ cells (t_(14)_ = 2.836, *p* = 0.0132, Figure 4D), and a decrease in the percentage of GFP + cells that expressed cFos [t_(14)_ = 4.885, *p* = 0.0002, Figure 4E]. We then performed the same comparisons in VNS- and Sham stimulated rats that received retro-eGFP infusions into the PL (Figures 4F–J). Separate unpaired t-tests found no difference in the number of GFP + cells [t_(12)_ = 0.6664, *p* = 0.5177, Figure 4H], but there was an overall decrease in cFos+ cells in VNS-treated rats [t_(12)_ = 2.653, *p* = 0.0211, Figure 4I], and an increase in the percentage of GFP + cells that expressed cFos in VNS-treated rats [t_(12)_ = 2.552, *p* = 0.0254, Figure 4J]. Taken together, these data suggest that pairing extinction with VNS selectively modulates neuronal activity in two pathways from the BLA to the IL and PL, respectively, in an opposite manner.

### 3.5 Effect of VNS on ventral hippocampal projections to the mPFC

**Figure 5:**
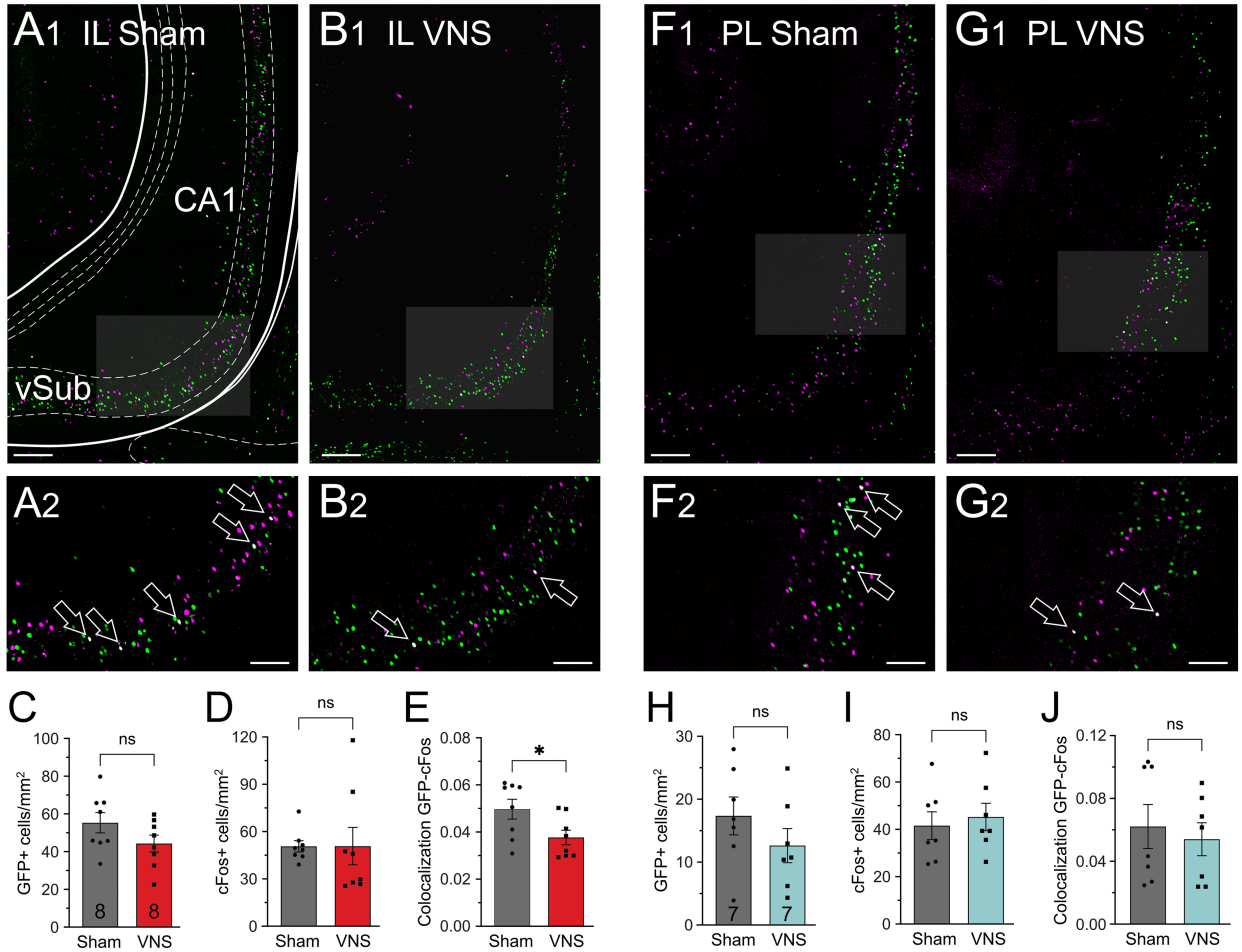
VNS modulates cFos expression following reinstatement differentially in IL- and PL-projecting neurons of the ventral hippocampus (vHPC). **(A,B)** GFP-positive cells (green) following retro-AAV infusion into the IL and cFos (pink) in the vHPC of rats receiving either Sham-stimulation (*n* = 8), **(A)** or VNS (*n* = 8) during extinction, **(B)**. **(A**_**2**_**,B**_**2**_**)** Arrows indicate GFP + cells colocalized with cFos (white). **(C)** VNS and Sham-stimulated rats did not differ in the number of GFP + cells in the vHPC. **(D)** VNS did not significantly affect overall cFos expression in the vHPC. **(E)** VNS also reduced cFos expression in IL-projecting (GFP+) neurons. **(F,G)** GFP-positive cells following infusion of the retro-AAV into the PL and cFos in the vHPC of rats receiving either Sham-stimulation (*n* = 7), **(F)** or VNS (*n* = 7) during extinction. **(H)** VNS and Sham-stimulated rats did not differ in the number of GFP + cells in the vHPC. **(I)** VNS did not affect overall vHPC cFos expression. **(J)** VNS did not alter cFos labeling in PL-projecting neurons. Scale bars represent 200 μm in **(A**_**1**_**,B**_**1**_**,F**_**1**_**,G**_**1**_**)**, and 100 μm in **(A**_**2**_**,B**_**2**_**,F**_**2,**_**G**_**2**_**).** *p* values are (*) < 0.05.

In rats that received AAV infusions of retro-eGFP into the IL we measured the number of cFos+ cells in the ventral subiculum and CA1 region of the vHPC as an indicator of cellular activity during cue-induced reinstatement in Sham-stimulated rats (*n* = 8) and rats given VNS (*n* = 8) (Figures 5A–E). Similarly, we measured the colocalization of IL-projecting (GFP+) neurons with cFos as a measure of activation of this pathway. Separate unpaired t-tests found no difference in the number of GFP + cells [t_(14)_ = 1.574, *p* = 0.1377, Figure 5C], no difference in the total number of cFos+ cells [t_(14)_ = 0.01091, *p* = 0.9914, Figure 5D], and a decrease in the percentage of GFP + cells that expressed cFos [t_(14)_ = 2.303, *p* = 0.0371, Figure 5E] between Sham- and VNS-treated rats.

Next, we performed the same analyses in rats that received AAV infusions of retro-eGFP into the PL. We compared eGFP and cFos expression, as well as their colocalization between rats that received Sham stimulation (*n* = 7) or VNS (*n* = 7) (Figures 5F–J). Separate unpaired t-tests found no difference in the number of GFP + cells [t_(12)_ = 1.166, *p* = 0.2661, Figure 5H] and no difference in cFos+ cells [t_(12)_ = 0.4423, *p* = 0.6661, Figure 5I]; and there was no difference in the percentage of GFP + cells that expressed cFos in VNS treated rats [t_(12)_ = 0.4659, *p* = 0.6497, Figure 5J]. These results indicate pathway-specific modulation of the projection from the vHPC to the IL by extinction paired with VNS.

### 3.6 VNS modulation of mPFC PVI activity during drug-seeking

**Figure 6:**
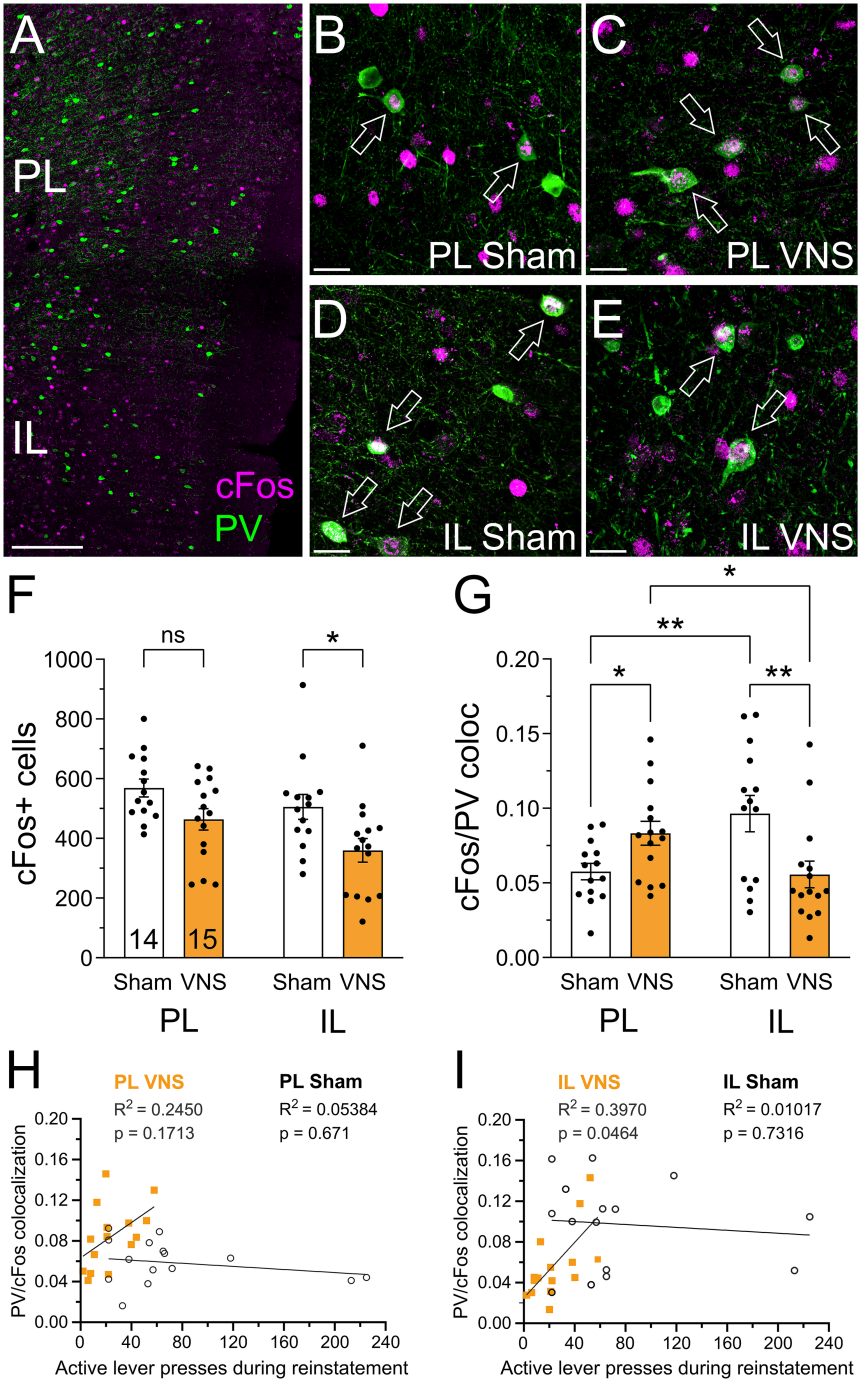
VNS affects activity of parvalbumin-positive interneurons (PVI) in the mPFC during drug-seeking. **(A)** cFos-positive cells (pink) and PVI (green) in the PL and IL. **(B,C)** Examples of cFos+ and parvalbumin+ cells in the PL of Sham- **(B)**, and VNS-stimulated rats **(C)**. **(D,E)** cFos+ and parvalbumin+ cells in the IL of Sham- **(D)**, and VNS-stimulated rats **(E)**. Arrows indicate PVI colocalized with cFos (white). **(F)** VNS decreased overall cFos expression in the IL, but not the PL. **(G)** Colocalization of cFos and PV in the PL and IL. In Sham treated animals PVI activity was reduced in the PL compared to IL, while in VNS treated animals PVI activity was greater in the PL compared to the IL. VNS increased the activity of PVI neurons in the PL but decreased cFos activity in PVI of the IL. **(H,I)** Correlation analyses between PV+/cFos+ colocalization and active lever presses during reinstatement in PVI in the PL **(H)** or IL **(I)** of Sham- and VNS-treated rats. In the IL of VNS-treated rats there was a significant negative correlation between cFos/PV colocalization and active lever presses. Scale bars represent 100 μm in **(A)** and 20 μm in **(B–E)**. *p* values are (*) < 0.05, and (**) < 0.01.

Projections from the vHPC and BLA to the mPFC make connections with PVIs that are powerful regulators of networks and behavioral output (McGarry and Carter, 2016; Marek et al., 2018; Aleman-Andrade et al., 2025). We stained slices of the mPFC for PV and cFos to determine activity of PVIs during cue-induced reinstatement in rats that received VNS (*n* = 15) or Sham-stimulation (*n* = 14) (Figure 6). We first quantified total cFos expression comparing PL to IL and Sham-stimulation to VNS. A two-way mixed-effects ANOVA with the factors treatment (Sham or VNS) and region (PL or IL) found significant effects for both factors [treatment, *F*_(1,27)_ = 7.670, *p* = 0.010; region, F_(1,27)_ = 9.98, *p* = 0.0039], but there was no interaction effect [F_(1,27)_ = 0.5619, *p* = 0.46; Figure 6F]. *Post hoc* testing with a Holm–Šídák multiple comparisons test showed no difference in overall cFos expression in the PL (*p* = 0.0960), while overall cFos decreased in the IL (*p* = 0.0304). We then examined the activation of PVIs in the PL and IL during cue-induced reinstatement by measuring colocalization of PVI and cFos immunofluorescence. A two-way mixed-effects ANOVA with factors of treatment (Sham or VNS) and region (PL or IL) found no significant effects for both factors, but a significant effect in the interaction of factors [treatment, F_(1,27)_ = 0.5732, *p* = 0.4556; region, F_(1,27)_ = 0.5152, *p* = 0.479; interaction, F_(1,27)_ = 17.97, *p* = 0.0002; Figure 6G]. Post hoc testing with a Holm–Šídák multiple comparisons test showed that in Sham-treated rats PVI activity was lower in the PL compared to the IL (*p* = 0.0075), while in VNS-treated rats the PL showed greater cFos activity in PVIs compared to the IL (*p* = 0.0345). In the PL VNS increased PVI activity (*p* = 0.0474), but in the IL VNS decreased PVI activity (*p* = 0.0075). We next examined the relationship between active lever presses during cue-induced reinstatement and the activation of PVIs with four separate linear regression analyses and used the Holm–Šídák method to correct for multiple comparisons. In the PL there was no significant association between lever pressing and PVI activation in both Sham (*p* = 0.6710) and VNS (*p* = 0.1713) treated rats (Figure 6H). In the IL there was no significant association between lever presses and PVI activation in Sham treated rats (*p* = 0.7316); however, in VNS-treated rats PVI activity had a significant negative association with lever presses during cue-induced reinstatement (*p* = 0.0464). These results indicate a VNS induced reduction in IL PVI activity in rats that show reduced relapse-like behavior.

## 4 Discussion

VNS can modulate cortical and subcortical circuits to improve extinction and reduce drug-seeking (Childs et al., 2017; Driskill et al., 2024). Here, we examined VNS-induced changes in cellular activity during drug-seeking in three brain areas that project to the IL and PL subregions of the mPFC which are important for drug seeking and the expression of extinction memories. We measured cFos expression in the PVT, BLA and vHPC, following cue-induced reinstatement, and we used retrograde labeling of projections to the IL and PL, respectively, to measure pathway-specific activation and modulation by VNS. We found that pairing extinction with VNS reduced reinstatement as previously described (Childs et al., 2017, 2019; Driskill et al., 2024). VNS altered activity in projections from the vHPC, PVT and BLA to the mPFC in a pathway-specific manner. In addition, VNS differentially altered activity of PVIs in the IL and PL, which are targets of projections from the BLA and vHPC.

### 4.1 Reduction of drug seeking by VNS

Exposure to drug-associated cues or stress induces craving and relapse in abstinent patients with substance use disorders (SUD). Extinction forms new associations that compete with these triggers to inhibit drug seeking; however, extinction is often not sufficient to reduce cue-reactivity and prevent relapse (Conklin and Tiffany, 2002), potentially because the brain areas required for extinction learning themselves become dysregulated by chronic drug use (Fowler et al., 2007; Stephens and Duka, 2008; Liu et al., 2009; Nic Dhonnchadha and Kantak, 2011).

Vagus nerve stimulation enhances activity-dependent plasticity by engaging neuromodulatory systems that bias active circuits toward adaptive synaptic modification (Hassert et al., 2004; Dorr and Debonnel, 2006; Roosevelt et al., 2006; Manta et al., 2009; Furmaga et al., 2011; Nichols et al., 2011; Manta et al., 2013), thereby amplifying learning-related plasticity when paired with behavioral training (Zuo et al., 2007; Engineer et al., 2011; Porter et al., 2012; Pena et al., 2014; Childs et al., 2017; Hulsey et al., 2019; Olsen et al., 2022). When combined with extinction training, VNS facilitates consolidation of extinction-related synaptic changes and reorganizes functional connectivity within limbic learning networks, including the amygdala and hippocampus (Cao et al., 2017; Childs et al., 2017; Hachem et al., 2018; Driskill et al., 2024). At the circuit level, VNS reshapes cocaine-induced maladaptive plasticity in a pathway-specific manner. Cocaine exposure increases baseline synaptic efficacy and promotes potentiation in the basolateral amygdala–infralimbic (BLA–IL) pathway, whereas pairing extinction with VNS normalizes synaptic strength and strengthens IL control over BLA output, consistent with a focusing of activity onto extinction-relevant ensembles (Childs et al., 2017; Arezoomandan et al., 2025). In parallel, VNS prevents cocaine-associated LTD in the prelimbic–nucleus accumbens (NAc) core pathway while selectively enhancing baseline synaptic strength and LTP in the infralimbic–NAc shell pathway, reinforcing cortical output that suppresses drug seeking (Arezoomandan et al., 2025). These effects align with evidence that relapse behavior is driven by projection-defined neuronal subpopulations rather than global activity changes (Bossert et al., 2011; Cruz et al., 2014; Moorman and Aston-Jones, 2015; Augur et al., 2016; McGlinchey et al., 2016; Warren et al., 2019; Forget et al., 2022; Zinsmaier et al., 2022; Thibeault et al., 2025). At the molecular level, VNS engages convergent plasticity-related signaling programs, including increased BDNF–TrkB signaling, enhanced expression of immediate early genes such as Arc and cFos, and upregulation of synaptic plasticity proteins including GluN2B and CaMKII (Biggio et al., 2009; Furmaga et al., 2012; Shen et al., 2012; Sanders et al., 2019; Olsen et al., 2022). In the context of drug-seeking, the TrkB-dependent synaptic adaptations restore infralimbic glutamatergic transmission and reduce relapse-like behavior (Driskill et al., 2024).

### 4.2 The role of the mPFC in drug-seeking

To further examine VNS-induced changes in networks that are important for the extinction and cue-induced reinstatement of drug-seeking, we focused on inputs to the mPFC.

Within the mPFC, the PL is often associated with the maintenance of drug-seeking behavior under conditions where cues are predictive of reward. The PL drives reinstatement via its projection to the NAc core. In contrast, the IL has been implicated in the suppression of drug-seeking, by aiding the formation and expression of extinction behaviors (Killcross and Coutureau, 2003; Di Ciano et al., 2007; Peters, LaLumiere et al., 2008; Peters, Vallone et al., 2008; LaLumiere et al., 2010; Van den Oever et al., 2013; Augur et al., 2016; Gutman et al., 2017; Muller Ewald et al., 2019; Nett et al., 2023). However, because frontal cortical areas are strongly interconnected, information about actions, emotions, and stimuli is available to all prefrontal regions (Euston et al., 2012; Anastasiades and Carter, 2021) and a number of studies have indicated overlapping roles for the PL and IL in reward seeking (Moorman and Aston-Jones, 2015; Gentry and Roesch, 2018; Madangopal et al., 2021), as reviewed in Howland et al. (2022).

Here, we focused on inputs from the PVT, BLA and vHPC that are important in appetitive associative learning and contextual processing. The goal was to determine whether neurons from these areas that send direct projections to the PL or IL, respectively, are selectively activated during reinstatement and whether pairing extinction training with VNS alters these activation patterns.

### 4.3 VNS modulation of PVT projections to the mPFC

The PVT occupies a strategic position within relapse-relevant circuitry, integrating arousal and motivational signals and relaying them to cortical and striatal targets that regulate reward seeking. Converging evidence indicates that PVT activity contributes to reinstatement-like behavior, although its precise role depends on the reinstatement trigger, physiological state, and projection target. Pharmacological inactivation of the PVT reduces cue-induced reinstatement of cocaine seeking (Kuhn et al., 2018), and intra-PVT manipulations that disrupt local signaling attenuate cocaine-primed reinstatement (James et al., 2010); related work further shows that transient inactivation of the pPVT blocks cocaine-seeking behavior (Matzeu et al., 2015), and that chemogenetic inhibition of PL neurons projecting to pPVT reduces both context-induced cocaine seeking after abstinence and cue-induced reinstatement after extinction (Giannotti et al., 2018). In addition, the PVT is required for context-induced renewal of extinguished reward seeking (Hamlin et al., 2009). However, dissecting the role of the PVT in reward seeking is complicated by a pronounced functional and anatomical heterogeneity along its antero-posterior axis, that shows graded specialization rather than strict subnuclear segregation (Li and Kirouac, 2012). Consistent with this, broad manipulations that span multiple PVT subregions can yield mixed or even opposing behavioral outcomes because they collapse functionally distinct ensembles and projection channels. Anatomical evidence indicates that aPVT and pPVT exhibit biased projection patterns, with aPVT preferentially targeting dorsomedial NAc shell and ventral subiculum and pPVT projecting more strongly to ventromedial NAc shell and structures of the extended amygdala (Vertes and Hoover, 2008; Li and Kirouac, 2012). Recent work in mice has also identified genetically and physiologically distinct PVT cell populations that differentially engage mPFC circuits and participate in arousal-modulated thalamo–corticothalamic loops: so-called Type I neurons are largely restricted to the pPVT and preferentially project to PL, while many Type II neurons reside in the aPVT and predominantly project to IL (Gao et al., 2020); Type I neurons were activated by aversive stimuli and inhibited by rewarding stimuli, whereas Type II neurons were inhibited by salient stimuli regardless of valence, suggesting that PVT-mPFC interactions can modulate cortical control processes and cue impact rather than encoding reward value per se (Gao et al., 2020). In the present study, we separately examined the effects of VNS on projections from the aPVT and pPVT to the mPFC. Pairing extinction training with VNS produced projection-specific changes in PVT recruitment during cue-induced reinstatement: VNS increased cFos expression selectively in aPVT neurons projecting to IL and in pPVT neurons projecting to PL, without altering overall cFos levels within either PVT subregion, suggesting that VNS does not globally suppress or enhance PVT activity but instead redistributes thalamic engagement across defined prefrontal output pathways. Functionally, enhanced recruitment of aPVT➔IL neurons following VNS may reflect greater engagement of thalamo-mPFC circuitry that supports extinction-related behavioral control during cue exposure. Conversely, increased recruitment of pPVT➔PL neurons in VNS-treated animals might reflect local PL circuit changes in inhibition because VNS simultaneously increased PVI activity in PL (see below). Importantly, the PVT is strongly linked to arousal and stress signaling, and manipulations of neuromodulatory inputs to pPVT can alter reinstatement in addiction-relevant settings (Matzeu and Martin-Fardon, 2020); because VNS engages ascending neuromodulatory systems and can alter anxiety-like behavior (Noble et al., 2019), a non-mutually exclusive interpretation is that VNS shifts global arousal state during reinstatement testing, thereby reshaping which PVT➔mPFC ensembles are recruited.

### 4.4 VNS modulation of BLA projections to the mPFC

The BLA is essential for cue-induced cocaine-seeking and relapse, serving as a critical hub that processes drug-associated memories and drives cocaine-seeking behavior, particularly in response to drug-associated cues. Furthermore, the BLA plays a complex dual role in extinction learning, being necessary for both the formation of cocaine-cue associations and their subsequent extinction (Di Ciano and Everitt, 2004; Fuchs et al., 2006; Stefanik and Kalivas, 2013; Pelloux et al., 2018). We have previously shown that VNS-induced suppression of cue-induced reinstatement following extinction correlates with a reduced expression of phospho-CREB in the BLA (Childs et al., 2017). Here we observed a similar global reduction in cFos signal in the BLA of VNS-treated rats, suggesting decreased recruitment of BLA ensembles during reinstatement. This reduced recruitment of BLA neurons may reflect altered encoding of cue-reward associations (Fuchs et al., 2006; Fuchs et al., 2009) or reduced motivational drive when exposed to triggering stimuli (Servonnet et al., 2020).

The BLA-mPFC pathway is integral in assigning emotional salience to drug-associated cues, facilitating the formation of strong drug-context associations that sustain addictive behaviors (Kantak et al., 2002; Kalivas and McFarland, 2003; McLaughlin and See, 2003; Di Ciano and Everitt, 2004; Peters, Vallone et al., 2008; Stefanik and Kalivas, 2013). Importantly, previous studies have shown that PL-innervating and IL-innervating neurons in BLA have different molecular markers and functions, with PL-innervating neurons encoding predominantly negative valence, while IL-projecting neurons mediate positive behaviors (Kim et al., 2016). Importantly, intermingled subpopulations of BLA projection neurons that target the PL and the IL, respectively, are differentially recruited during either the expression of fear (PL) or during fear extinction (IL) (Senn et al., 2014). We hypothesized that the BLA regulates mPFC activity in a similar manner during the extinction of drug-seeking and that VNS would enhance activity in the IL-projecting BLA neurons to facilitate or consolidate extinction to reduce responding during cue-induced reinstatement. Alternatively, VNS might weaken inputs from the BLA to the PL which drive drug-seeking (Stefanik and Kalivas, 2013).

Surprisingly, we observed the opposite activation pattern in VNS-treated animals: PL-projecting neurons showed increased cFos activity after cue-induced reinstatement, whereas IL-projecting neurons in the BLA showed reduced cFos expression. One potential explanation for this unexpected result is that the net influence of glutamatergic BLA inputs to the mPFC is predominantly inhibitory. BLA afferents preferentially target parvalbumin- and somatostatin-expressing interneurons, which provide strong feedforward inhibition onto IL projection neurons, including cortico-amygdala neurons that project back to the BLA (Gabbott et al., 2006; Dilgen et al., 2013; McGarry and Carter, 2016). Thus, reduced cFos expression in BLA➔IL neurons may reflect a shift in the balance of excitatory drive onto IL networks, potentially favoring IL ensembles associated with extinction expression. Similarly, enhanced feedforward inhibition within the BLA➔PL pathway following VNS during extinction training could suppress PL activity and reduce drug-seeking behavior. Together, the anatomical and functional organization of BLA projections to the mPFC provides a plausible circuit-level framework for how opposing VNS-induced modulation of these pathways may promote extinction and suppress relapse. However, it is important to note that while our cFos data indicate pathway-specific modulation within the BLA it does not directly address the functional consequences of this modulation in the mPFC itself.

### 4.5 VNS modulation of vHPC projections to the mPFC

The hippocampus integrates contextual, emotional, and mnemonic information, and reactivation of these representations can drive craving and relapse (Neisewander et al., 2000; Kalivas and McFarland, 2003; Atkins et al., 2008; Lasseter et al., 2010; Goode and Maren, 2019). Direct hippocampal projections to the medial prefrontal cortex (mPFC) arise predominantly from the vHPC (Jay and Witter, 1991; Cenquizca and Swanson, 2007; Aleman-Andrade et al., 2025). Consistent with a causal role in relapse, electrical stimulation of the ventral subiculum - the principal output of the vHPC - reinstates extinguished drug seeking, whereas inactivation of the vHPC or ventral subiculum attenuates both cue-induced and cocaine-primed reinstatement (Vorel et al., 2001; Sun and Rebec, 2003; Taepavarapruk and Phillips, 2003; Rogers and See, 2007). The vHPC–mPFC pathway is critical for behaviors that require integration of contextual and emotional information with executive control processes (Vertes, 2006). For example, context-induced renewal of heroin seeking selectively engages the vHPC➔infralimbic (IL) projection, and silencing this pathway prevents reinstatement, whereas vHPC➔prelimbic (PL) projections are not required (Bossert et al., 2016; Wang et al., 2018). In addition, vHPC inputs to the mPFC also support the consolidation and retrieval of extinction memories, contributing to suppression of conditioned drug responses (Castilla-Ortega et al., 2016; Kutlu and Gould, 2016). The bidirectional organization of vHPC–mPFC circuitry enables flexible behavioral regulation across changing contexts, making this pathway a plausible target for VNS-mediated modulation of synaptic plasticity during extinction learning.

In our data we found no differences between VNS- and Sham-stimulated rats in the total number of cFos+ cells in the vHPC, indicating that pairing extinction with VNS did not affect overall activity of the vHPC during reinstatement. However, we observed pathway-specific alterations in the number of cFos+ vHPC cells, such that in VNS-treated rats IL-projecting cells (but not cells projecting to the PL) showed a selective reduction of cFos expression. Previous work has shown that activation of the vHPC➔IL pathway engages strong inhibition of IL outputs, preventing extinction mechanisms of both drug-seeking (Bossert et al., 2016, Wang et al., 2018) and fear renewal (Marek et al., 2018). Thus, reduced cFos recruitment in vHPC➔IL neurons may reflect decreased engagement of this pathway during reinstatement, potentially biasing IL network dynamics toward extinction-related processing. In contrast, the lack of VNS modulation of the vHPC➔PL projection could either further support a functional dissociation between vHPC projections to the IL and PL as previously suggested (Bossert et al., 2016, Wang et al., 2018), or it may reflect the relatively weaker innervation from the vHPC to the PL (Wang et al., 2016; Goode and Maren, 2019).

### 4.6 VNS modulation of PVI in the mPFC

Afferents from both the vHPC and the BLA directly contact pyramidal cells and interneurons in the mPFC. As outlined above projections onto PVI and somatostatin-expressing interneurons have been shown to exert strong inhibitory effects on the functions of the PL and IL (Tierney et al., 2004; McGarry and Carter, 2016; Abbas et al., 2018; Liu and Carter, 2018; Marek et al., 2018; Sun et al., 2019; Yang et al., 2021; Sanchez-Bellot et al., 2022; Aleman-Andrade et al., 2025). Importantly, PVI in the PL and IL receive inputs from largely different neurons within the same brain areas (Sun et al., 2019). Activation of PL PVI supports the transition from a cue-driven to a flexible behavioral state, thus facilitating extinction of reward-seeking behavior (Sparta et al., 2014). We examined VNS-induced changes in cFos expression in the mPFC, as well as cFos colocalization with PVI in the PL and IL to determine if VNS may alter PVI activity to facilitate extinction and reduce reinstatement. Consistent with our previous findings using the activitiy marker pCREB (Childs et al., 2017), we found an overall reduction in cFos activity in the IL with no significant change in the PL. Thus, while cFos expression can serve as a general marker for VNS-induced changes in activity in the mPFC, it is by itself not sufficient to distinguish potentially different roles of multiple overlapping networks for drug-seeking or extinction in the IL and PL. However, when we examined the colocalization of cFos with PVI we found that VNS caused opposing, area-specific changes: PVI in the PL showed increased cFos expression, consistent with enhanced engagement of local inhibitory interneuron networks, which may shift PL ensemble dynamics away from relapse-promoting activity. This increase in cFos expression in PVI is also consistent with an enhanced drive from upstream BLA in VNS animals (Figure 4J). In contrast, PVI in the IL showed less cFos activity, indicating VNS-induced disinhibition of IL networks. This is also consistent with our finding that VNS reduced activity in the projections from the vHPC and BLA, and previous findings outlined above which show that inactivation of IL-projecting neurons prevents reinstatement of fear and drug-seeking (Marek et al., 2018; Wang et al., 2018; Binette et al., 2023). These effects are also highlighted by a significant negative association between PVI activity and lever presses during reinstatement in VNS treated rats. Taken together, these findings suggest that VNS reshapes mPFC ensemble recruitment during reinstatement, biasing PL circuits toward greater interneuron engagement while favoring IL ensembles associated with extinction expression. These effects may be driven by VNS-induced changes in projections from the vHPC and the BLA.

## 5 Limitations

Our findings have important limitations: Our results are correlational and do not establish causal relationships between VNS-induced changes in PVI activity, alterations in upstream projections to the mPFC, and behavioral outcomes. Because cFos reflects transcriptional activation within recently engaged neuronal ensembles, our findings indicate relative pathway recruitment rather than direct changes in firing rate or synaptic inhibition. Furthermore, it is unclear how many PVI in the mPFC receive inputs from the BLA and vHPC and what the functional significance of these inputs is. In addition, projections from the vHPC and BLA innervate other interneuron populations beyond PVIs, including somatostatin and vasoactive intestinal peptide interneurons (McGarry and Carter, 2016; Sun et al., 2019). These interneuron subtypes powerfully regulate mPFC function and can themselves inhibit PVIs (Xu et al., 2019; Yang et al., 2021). Therefore, future studies using projection-specific optogenetic or chemogenetic approaches must establish direct causal links between VNS-induced changes in mPFC projections and the activity of PVIs and other interneuron populations in regulating drug-seeking behavior. Another limitation is that our study used only male rats and therefore we cannot exclude potential sex differences in the mechanisms through which VNS modulates extinction learning. This concern is somewhat reduced by two recent observations: (1) VNS modulates extinction learning and cue-induced reinstatement in both male and female rats. (2) VNS-induced changes in long-term synaptic plasticity in the pathway from the BLA to the IL show no sex-dependent differences (Arezoomandan et al., 2025).

## 6 Summary

Our work demonstrates that vagus nerve stimulation (VNS) paired with extinction training reduces drug-seeking behavior by selectively modulating prefrontal cortex circuits. We found that VNS differentially affects PVI, increasing inhibitory activity in the PL (which drives drug-seeking) while decreasing it in the IL (which supports extinction). VNS also altered pathway-specific projections from the PVT, vHPC, and BLA, creating enhanced extinction circuits while suppressing relapse-driving networks. Our findings reveal how neuromodulation can precisely target competing neural circuits in addiction, suggesting VNS could serve as an adjunctive therapy for substance use disorders by strengthening the brain’s natural recovery mechanisms while weakening cue-driven relapse pathways.

## Data Availability

The original contributions presented in the study are included in the article/supplementary material, further inquiries can be directed to the corresponding author/s.
